# Diverging trends in primary and revision arthroplasty in Germany: projections and implications for healthcare delivery for the next ten years

**DOI:** 10.1007/s00264-026-06883-3

**Published:** 2026-06-12

**Authors:** Dirk Linke, Gautier Beckers, Bernd Kladny, Susanne Mayer-Wagner, Maximilian Rudert, Boris Michael Holzapfel, Ioannis Stratos

**Affiliations:** 1 Department of Orthopaedics and Trauma Surgery, Ludwig-Maximilians-Universität München, Munich, Germany; 2 German Society for Orthopaedics and Orthopaedic Surgery, Berlin, Germany; 3 Orthopaedic Hospital König-Ludwig-Haus, University of Würzburg, Wurzburg, Germany

**Keywords:** Hip Arthroplasty, Knee arthroplasty, Revision surgery, Healthcare centralization, Demographic change

## Abstract

**Purpose:**

To analyze nationwide trends, demographic changes, and future projections of primary and revision hip and knee arthroplasty in Germany.

**Methods:**

Nationwide data from the German Federal Statistical Office and hospital quality reports (2015–2024) were analyzed regarding procedure volumes, age distribution, hospital stay, and structural care changes. Future case numbers until 2035 were projected using age-specific incidence rates and population forecasts.

**Results:**

Primary hip and knee arthroplasty volumes increased significantly, with annual growth rates of + 4,986 cases for hip (p = 0.005) and + 5,727 cases for knee arthroplasty (p = 0.028), whereas revision procedures remained stable (hip: p = 0.065; knee: p = 0.947). Age-stratified analyses demonstrated a shift toward older patients, with increasing proportions in age groups ≥ 60 years and declining shares among patients ≤ 50 years. Projections to 2035 indicate stable overall primary procedure numbers (+ 637 cases), with an increase in hip arthroplasty (+ 11,202 cases) and a decrease in knee arthroplasty (− 10,565 cases), while revision procedures are expected to rise by + 3,158 cases, particularly in patients > 75 years. Length of hospital stay decreased significantly across all procedures (− 0.38 to − 0.46 days per year; p < 0.001), and the number of hospitals declined markedly between 2019 and 2024 (− 19.2 to − 25.6 hospitals per year), reflecting increasing centralization of primary care, whereas revision procedures remained widely distributed, also across low-volume centers.

**Conclusion:**

These findings reveal a growing mismatch between increasing demand, demographic changes, and current care structures, underscoring the need for targeted adaptations in arthroplasty care delivery.

## Introduction

Total joint arthroplasty is emerging as a major challenge for healthcare systems in the context of aging societies. In Germany, the number of primary hip and knee arthroplasty procedures has increased steadily over recent decades, driven by demographic changes and the progressive expansion of surgical indications [1], further influenced by the diagnosis-related group (DRG)–based hospital reimbursement system. Therefore, a substantial rise in revision procedures has long been anticipated, multiple previous national and international studies projecting a significant increase in revision burden in the coming years [2, 3]. This rising demand is accompanied by increasing healthcare expenditures and resource utilization, placing growing pressure on system capacity and long-term sustainability. As a consequence, structural changes in healthcare delivery are underway. Moreover, evidence suggests that higher hospital case volumes are associated with improved outcomes and lower revision rates [4]. Accordingly, regulatory measures such as minimum case volume thresholds and increasing economic pressures are promoting centralization of care toward higher-volume institutions.

At the same time, advances in perioperative management have led to a continuous reduction in length of hospital stay, reflecting improvements in efficiency and resource utilization [5]. However, the interaction between demographic developments, procedural trends, healthcare structures, and resource allocation remains insufficiently understood, particularly in light of substantial heterogeneity between national healthcare systems. Previous projections of arthroplasty demand have primarily relied on general population forecasts without incorporating age-specific trends, changes in hospital structures, or evolving patterns of care delivery. A comprehensive evaluation of future healthcare needs therefore requires an integrated analysis that combines demographic projections with procedure-specific trends and structural characteristics of the healthcare system under investigation.

The aim of the present study was to provide a comprehensive, age-stratified analysis of procedural care for hip and knee arthroplasty surgeries in Germany, including temporal trends in procedure volumes, length of hospital stay, and structural changes in hospital care delivery, and to project future demand up to the year 2035 based on national population forecasts.

## Materials and methods

### Age-stratified analysis of arthroplasty procedures over time

An age-stratified analysis of procedure volumes was conducted for the period from 2015 to 2024. Cases were categorized into predefined age groups (< 50 years, 50–60 years, 60–75 years, and > 75 years) to evaluate temporal trends across different patient age groups. The analysis was based on data from the German Federal Statistical Office (Destatis), specifically the dataset “Hospital Statistics on Operations and Procedures of Inpatients (number of procedures by code).” Procedures were identified using the German Operation and Procedure Classification System (OPS). Primary total hip arthroplasty (THA) was defined using OPS codes 5–820.x, primary total knee arthroplasty (TKA) using codes 5–822.x, revision hip arthroplasty using codes 5–821.x, and revision knee arthroplasty using codes 5–823.x, including all respective subcodes. The extracted data were subsequently processed and aggregated to annual values using pivot tables in Microsoft Excel (Microsoft Office 365, Microsoft Corp., Redmond, WA, USA). Datasets generated using this method include temporal trends in primary and revision arthroplasty volumes (2015–2024) and the age distribution of arthroplasty procedures over time (2015–2024).

### Projection methodology for 2035

The projection for the year 2035 was performed as follows: First, the incidence of primary total hip arthroplasty (THA), primary total knee arthroplasty (TKA), as well as revision procedures for hip and knee arthroplasties was determined for the year 2024. Subsequently, the population forecast for Germany in 2035, as provided by the German statistical offices of the federal states, which were subsequently harmonized and validated against the federal population projections, was used as the basis. Assuming constant incidence rates within the respective age groups (< 50 years, 50–60 years, 60–75 years, and > 75 years), the incidence rates at the district level obtained for 2024 were extrapolated to the projected population of 2035 in order to estimate the future number of procedures. Datasets generated using this method include the projected changes in arthroplasty volumes by age group (2024–2035).

### Length of hospital stay analysis

Data on length of hospital stay were obtained from the official statistical report “Diagnoses of Hospital Patients (case numbers and length of stay by diagnosis)” provided by the German Federal Statistical Office (Destatis). Relevant ICD-10 codes for primary hip and knee arthroplasty as well as revision procedures were extracted from pre-aggregated datasets covering the years 2015 to 2024. For selected indications, not all ICD subcategories were fully attributable to arthroplasty procedures; therefore, the corresponding length of stay values were included proportionally in the analysis. Relevant ICD-10 codes were defined to identify procedures of interest. For primary total hip arthroplasty (THA), the codes M16.0–M16.7, M16.9, and S72.0–S72.1 were included. For primary total knee arthroplasty (TKA), codes M17.0–M17.5 and M17.9 were used. Revision arthroplasty procedures were identified using the codes M00.0–M00.2, M00.8–M00.9, M86.0–M86.6, M86.8–M86.9, T84.0, T84.5, and Z96.6. The extracted data were subsequently processed and aggregated to annual values using pivot tables in Microsoft Excel. Based on these data, a dataset was generated to analyze trends in length of hospital stay by procedure type (2015–2024).

### Hospital quality report analysis

For the analyses of temporal trends in the number of hospital sites by procedure type (2019–2024) and in hospital site volume categories (2019–2024), data were obtained from hospital quality reports provided by the German Federal Joint Committee (G-BA), accessed via the official reference database and a licensed data provider. The datasets were available in structured XML format at the hospital site level and comprised multiple files per institution. Relevant procedure codes (OPS) were extracted. The data were processed and aggregated at the four-digit OPS level and subsequently grouped into predefined procedure categories (primary hip arthroplasty, primary knee arthroplasty, revision hip arthroplasty, and revision knee arthroplasty). For each hospital site, procedure volumes were summarized using pivot-based aggregation, and institutions were categorized into predefined volume groups. For primary arthroplasty, low hospital volume was defined as < 50 procedures per year, medium volume as 50–150, and high volume as > 150. For revision arthroplasty, low volume was defined as < 25 procedures per year, medium volume as 25–50, and high volume as > 50. Based on this classification, a matrix of hospital sites and case volume categories was constructed for further analysis. Based on this classification, datasets were generated to analyze temporal trends in the number of hospitals by procedure type (2019–2024) and trends in hospital volume categories over time (2019–2024).

### Statistical analyses and data visualization

Statistical analyses and data visualization were performed using RStudio (version 2025.09.2 + 418; R Foundation for Statistical Computing, Vienna, Austria). Temporal trends in procedure volumes and length of stay were assessed using linear regression models, complemented by descriptive analyses across age groups and hospital-related parameters. Future case numbers for 2035 were projected based on age-specific incidence rates from 2024 and official population forecasts at the district level, with results visualized using graphical methods implemented in R.

## Results

**Fig. 1:**
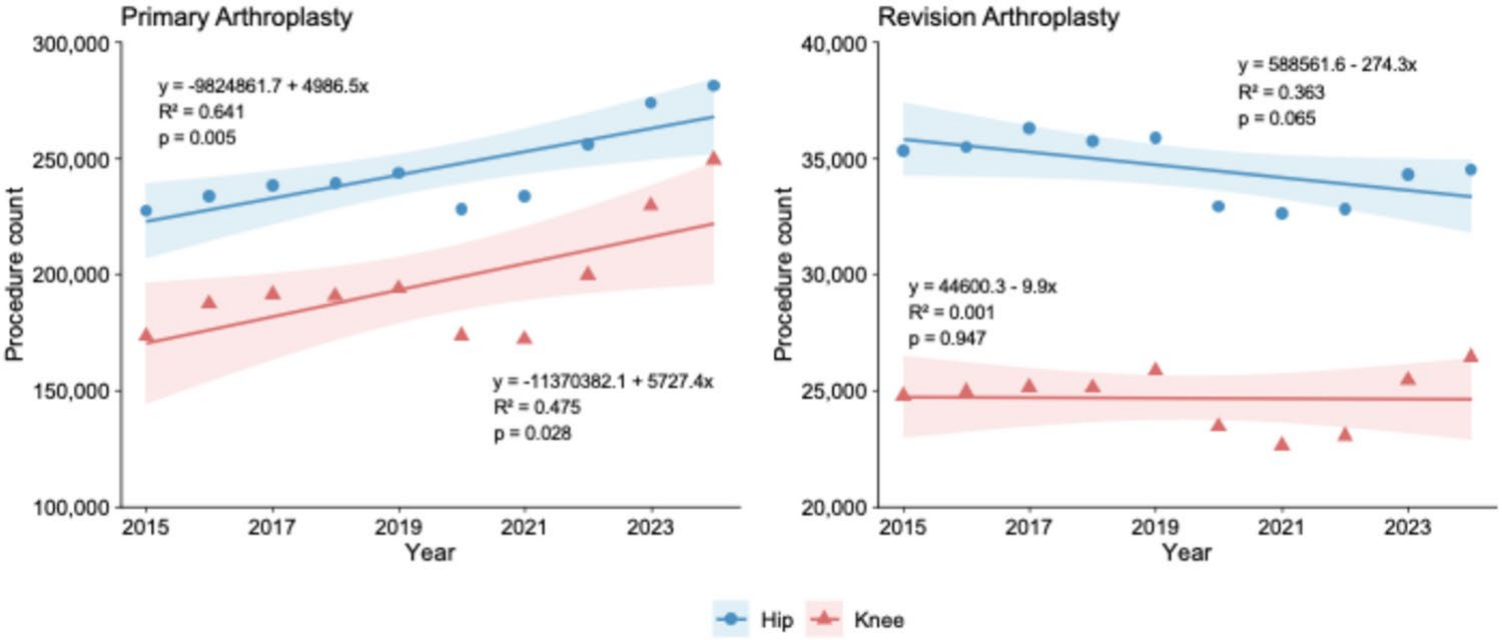
Temporal Trends in Primary and Revision Arthroplasty Volumes (2015–2024). Observed annual counts are shown as points. Linear regression lines with 95% confidence intervals (shaded) illustrate trends over time. Circles indicate hip arthroplasty and triangles indicate knee arthroplasty

The annual number of primary hip and knee arthroplasties in Germany showed a clear increasing trend over time (Fig. 1). Linear regression analysis demonstrated a significant rise in primary hip arthroplasty, with an average annual increase of approximately 4,986 procedures (95% CI: 1,941 to 8,032; p = 0.005). Similarly, primary knee arthroplasty increased significantly by about 5,727 procedures per year (95% CI: 817 to 10,637; p = 0.028). These findings indicate a steady and statistically significant growth in primary joint replacement procedures in Germany over the study period. In contrast, revision hip arthroplasty showed a slight decreasing trend; however, this change did not reach statistical significance (p = 0.065). Likewise, revision knee arthroplasty remained essentially constant over time, with no significant change observed (p = 0.947), indicating stable revision rates in Germany without a significant temporal change.

**Fig. 2:**
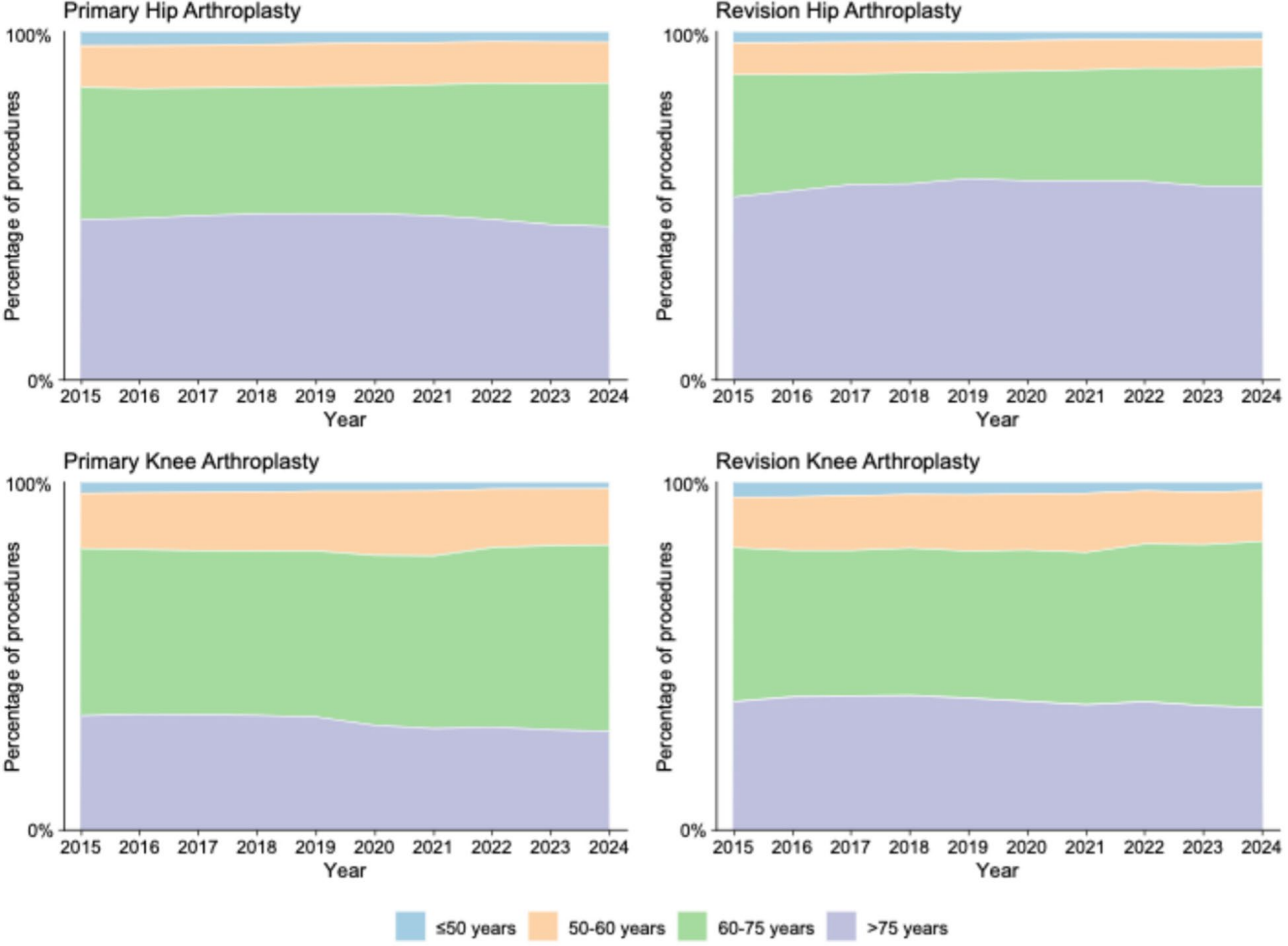
Age Distribution of Arthroplasty Procedures Over Time (2015–2024). Stacked area plots display the relative distribution of primary and revision hip and knee arthroplasty procedures across age groups over time. Each panel represents one procedure type and joint. Colored areas indicate the proportion of procedures performed in each age group (≤ 50, 50–60, 60–75, and > 75 years), expressed as percentages of the total annual volume. Changes in the composition of age groups over time can be visually assessed within each panel

The relative distribution of procedures across age groups changed over time for both primary and revision arthroplasty in Germany (Fig. 2, Table 1). In primary hip arthroplasty, the proportion of younger patients (≤ 50 years) showed a decreasing trend, while the share of patients aged 50–60, 60–75, and > 75 years increased significantly, indicating a shift toward older age groups. A similar pattern was observed for primary knee arthroplasty in Germany, where the proportion of younger patients (≤ 50 years) declined significantly, while the 50–60 and 60–75 year groups increased significantly over time. For revision arthroplasty, the distribution differed from primary procedures. In revision hip arthroplasty, the proportions of younger (≤ 50 years) and middle-aged patients (50–60 years) decreased significantly over time, while older age groups also showed a downward trend in hip revision surgeries per year, although without statistically significant changes. In revision knee arthroplasty, the proportion of younger patients (≤ 50 years) also decreased significantly, whereas the other age groups showed no significant temporal trends. Overall, these findings indicate a demographic shift toward older patients in primary arthroplasty in Germany, while revision procedures show a reduction in younger patient groups but otherwise relatively stable age distributions.

**Table 1:** Linear regression analysis by age group

Procedure Type	Age Group	Intercept	Slope (per year)	95% CI (Slope)	R^2^	p-value
Primary Hip Arthroplasty	≤ 50 years	326,992	− 158.00	[− 315.93; 0.57]	0.398	0.051
	50–60 years	− 887,278	454.00	[135.55; 772.68]	0.575	**0.011**
	60–75 years	− 5,960,633	2,998.00	[1,148.76; 4,846.79]	0.636	**0.006**
	> 75 years	− 3,303,833	1,692.00	[727.49; 2,656.96]	0.672	**0.004**
Primary Knee Arthroplasty	≤ 50 years	317,806	− 155.00	[− 254.53; − 55.24]	0.616	**0.007**
	50–60 years	− 1,990,361	1,002.00	[553.38; 1,450.44]	0.768	** < 0.001**
	60–75 years	− 8,718,827	4,365.00	[1,474.95; 7,256.02]	0.603	**0.008**
	> 75 years	− 979,000	515.00	[− 1,105.15; 2,134.84]	0.063	0.485
Revision Hip Arthroplasty	≤ 50 years	104,759	− 51.40	[− 69.79; − 33.03]	0.839	** < 0.001**
	50–60 years	163,640	− 79.50	[− 103.79; − 55.29]	0.877	** < 0.001**
	60–75 years	180,110	− 83.60	[− 246.53; 79.34]	0.149	0.271
	> 75 years	140,053	− 59.80	[− 262.84; 143.29]	0.054	0.516
Revision Knee Arthroplasty	≤ 50 years	109,021	− 53.60	[− 70.59; − 36.55]	0.868	** < 0.001**
	50–60 years	9,654	− 2.88	[− 52.09; 46.32]	0.002	0.896
	60–75 years	− 250,420	129.00	[− 63.06; 321.79]	0.231	0.160
	> 75 years	176,345	− 82.80	[− 219.25; 53.70]	0.196	0.199

Linear regression analyses were performed to evaluate temporal trends in procedure counts stratified by joint (hip and knee), procedure type (primary and revision arthroplasty), and age group (see Fig. 2). The intercept represents the estimated baseline value at year zero, while the slope indicates the annual change in the number of procedures within each subgroup. Ninety-five percent confidence intervals (CI) are provided for slope estimates. Model fit is described by the coefficient of determination (R^2^), and statistical significance is assessed using p-values. Trends are categorized as increasing or decreasing based on the direction of the slope

**Fig. 3:**
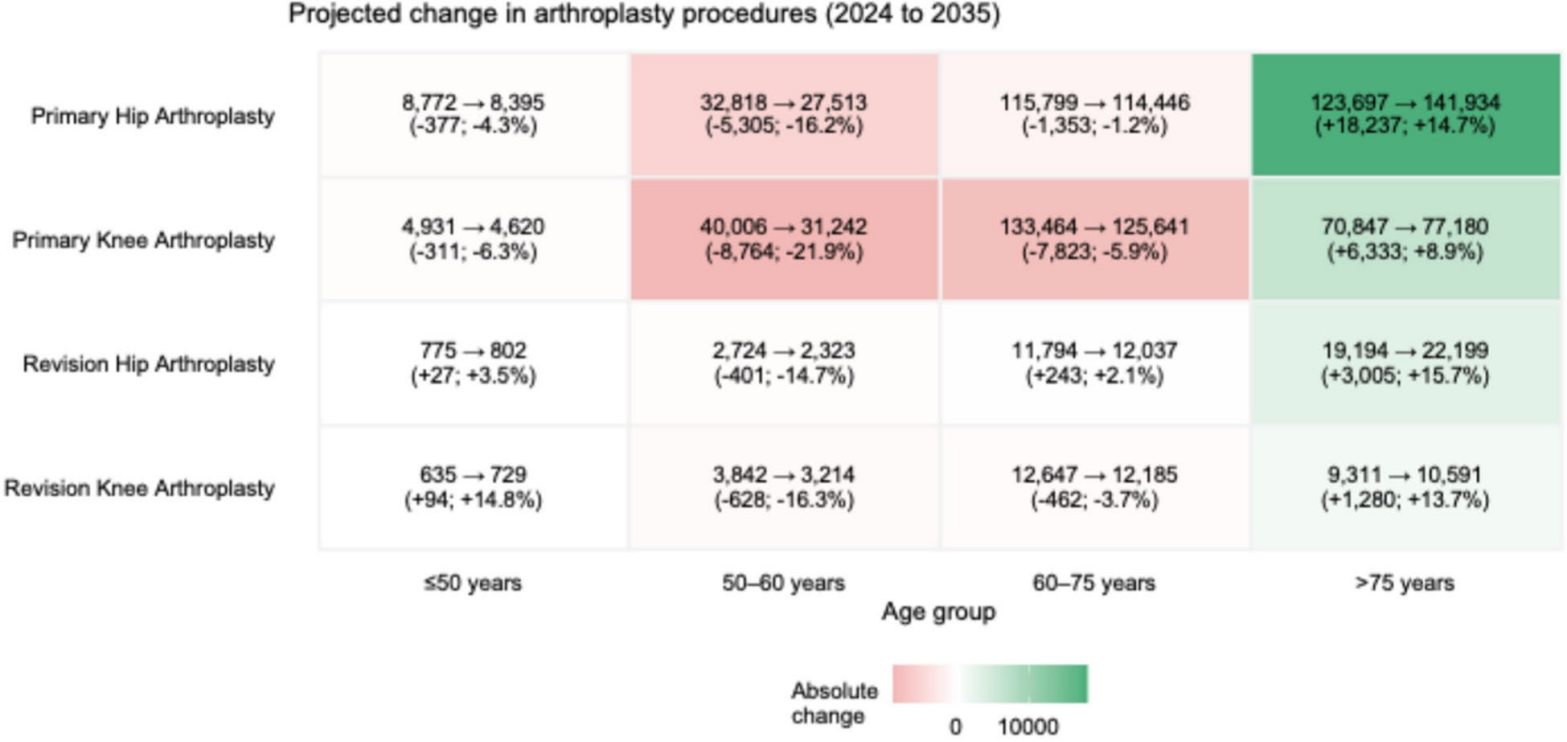
Projected Changes in Arthroplasty Volumes by Age Group (2024 to 2035). Heatmap showing projected absolute changes in arthroplasty procedures by age group and procedure type. Cells display counts for 2024 and 2035 (top) and absolute/relative change (bottom). Colors indicate direction and magnitude of change (red = decrease, green = increase)

Projected changes between 2024 and 2035 in Germany demonstrate distinct age-dependent trends across all procedure types (Fig. 3). In primary hip arthroplasty, the number of procedures is expected to decrease in younger and middle-aged patients (≤ 50 and 50–60 years), while remaining relatively stable in the 60–75 age group and increasing markedly in patients older than 75 years (+ 14.7%). A similar pattern is observed for primary knee arthroplasty in Germany, with pronounced declines in the 50–60 age group (− 21.9%) and moderate decreases in the 60–75 group, whereas procedures in patients > 75 years are projected to increase (+ 8.9%). For revision hip arthroplasty, projections indicate a decrease in the 50–60 age group (− 14.7%), while the ≤ 50, 60–75, and > 75 year groups are expected to increase, with the strongest relative growth in patients older than 75 years (+ 15.7%). In revision knee arthroplasty, a comparable pattern is observed, with decreases in the 50–60 and 60–75 age groups, contrasted by increases in younger patients (≤ 50 years, + 14.8%) and particularly in those > 75 years (+ 13.7%). Overall, between 2024 and 2035, the total number of primary arthroplasties in Germany is projected to remain largely stable (+ 637 cases), with an increase in primary hip procedures (+ 11.202 cases) and a decrease in primary knee procedures (− 10.565 cases). In contrast, revision procedures in Germany are expected to rise substantially (+ 3,158), driven by increases in both revision hip (+ 2,874 cases) and revision knee (+ 284 cases) arthroplasties, particularly in older age groups.

**Fig. 4:**
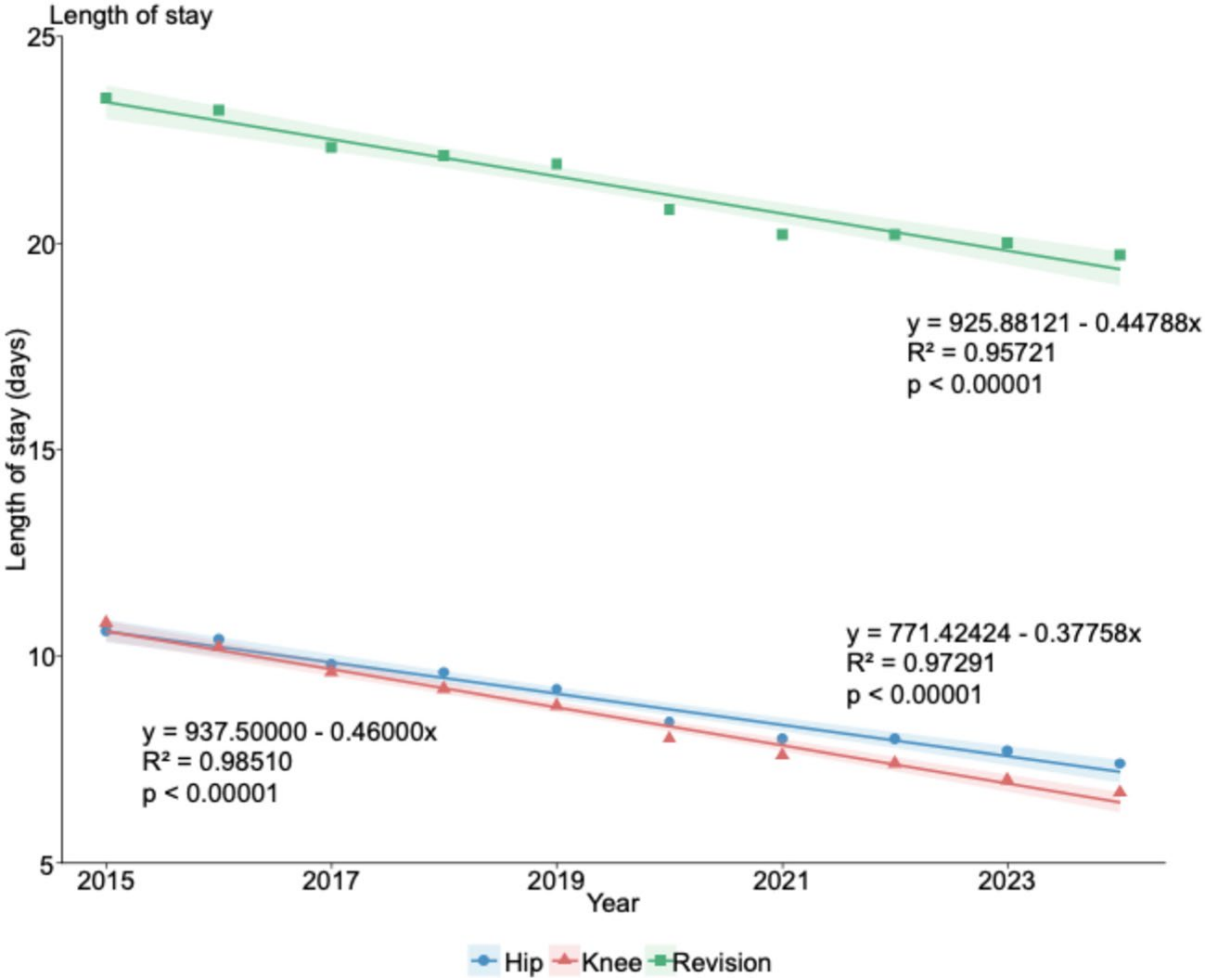
Trends in Length of Hospital Stay by Procedure Type (2015–2024). Observed annual length of stay values are shown as points. Linear regression lines with 95% confidence intervals (shaded) illustrate trends over time. Circles indicate hip arthroplasty, triangles indicate knee arthroplasty, and squares indicate revision hip and knee arthroplasty

A continuous decline in length of stay was observed across all procedure types in Germany over the study period. As shown in Fig. 4, linear regression analysis demonstrated a significant annual reduction of 0.38 days for primary hip arthroplasty (95% CI: − 0.43 to − 0.33; p < 0.001). Similarly, length of stay for primary knee arthroplasty in Germany decreased significantly by 0.46 days per year (95% CI: − 0.51 to − 0.41; p < 0.001). Revision arthroplasty procedures also showed a significant reduction, with an annual decrease of 0.45 days (95% CI: − 0.53 to − 0.37; p < 0.001). Extrapolation of the regression models to the year 2035 suggests a further reduction in length of stay to approximately 3.0 days for primary hip arthroplasty, 1.4 days for primary knee arthroplasty, and 14.4 days for revision procedures. While the projections for hip arthroplasty indicate a theoretical value, the overall trend clearly reflects ongoing improvements in perioperative care and efficiency toward shorter surgical stays for arthroplasties in Germany.

**Fig. 5:**
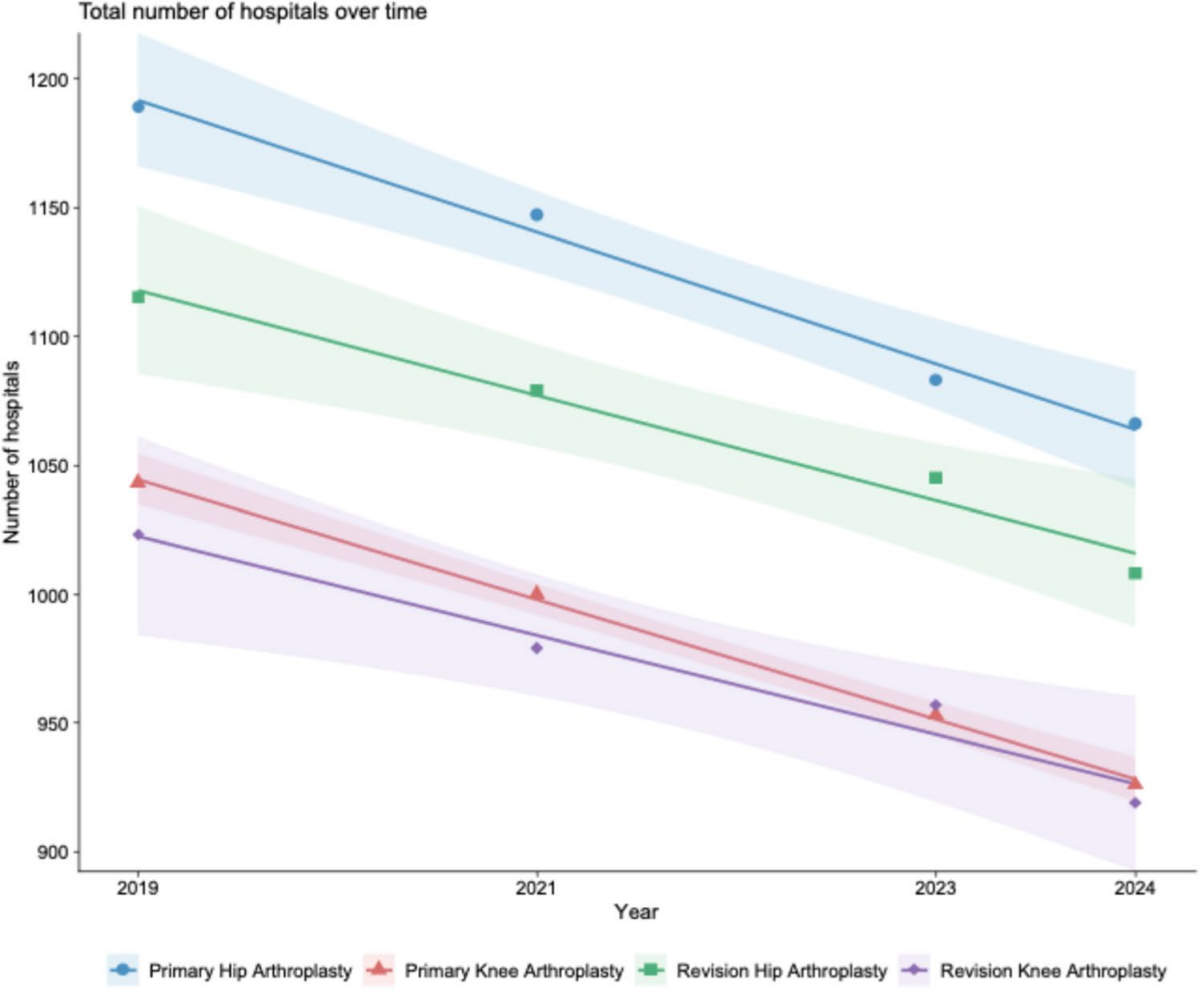
Temporal Trends in the Number of Hospitals by Procedure Type (2019–2024). Observed annual numbers of hospitals are shown as points. Linear regression lines with corresponding 95% confidence intervals (shaded areas) illustrate temporal trends between 2019 and 2024 (see also Table 2). Colors differentiate procedure types: blue indicates primary hip arthroplasty, red primary knee arthroplasty, green revision hip arthroplasty, and purple revision knee arthroplasty. Distinct point shapes further distinguish the procedure categories. For further details about the linear model see Table 2

The number of hospitals providing surgical care for arthroplasties in Germany decreased consistently between 2019 and 2024 across all procedure types (Fig. 5, Table 2). Primary hip and knee arthroplasty showed significant annual reductions of 25.6 and 23.3 hospitals, respectively (both p ≤ 0.005). Similarly, revision hip and knee arthroplasty declined by 20.4 and 19.2 hospitals per year (p ≤ 0.019). The high R^2^ values indicate a strong linear trend. Overall, these results demonstrate a significant and consistent consolidation of surgical care for arthroplasties over time in Germany.

**Table 2:** Linear regression analysis of total hospital counts

Procedure Type	Intercept	Slope (per year)	95% CI (Slope)	R^2^	p-value
Primary Hip Arthroplasty	52,899	−25.6	[−33.31; −17.91]	0.990	**0.005**
Primary Knee Arthroplasty	48,063	−23.3	[−26.20; −20.37]	0.998	** < 0.001**
Revision Hip Arthroplasty	42,353	−20.4	[−30.13; −10.72]	0.976	**0.012**
Revision Knee Arthroplasty	39,828	−19.2	[−30.70; −7.74]	0.963	**0.019**

Linear regression analyses were performed to assess temporal trends in the total number of hospitals across procedure types (complementary to Fig. 5). The intercept represents the estimated baseline value at year zero, while the slope indicates the annual change in the number of hospitals. Ninety-five percent confidence intervals (CI) are provided for the slope estimates. Model fit is described by the coefficient of determination (R^2^), and statistical significance is assessed using p-values. All models demonstrate a consistent decline in hospital numbers over time across both primary and revision arthroplasty procedures

**Fig. 6:**
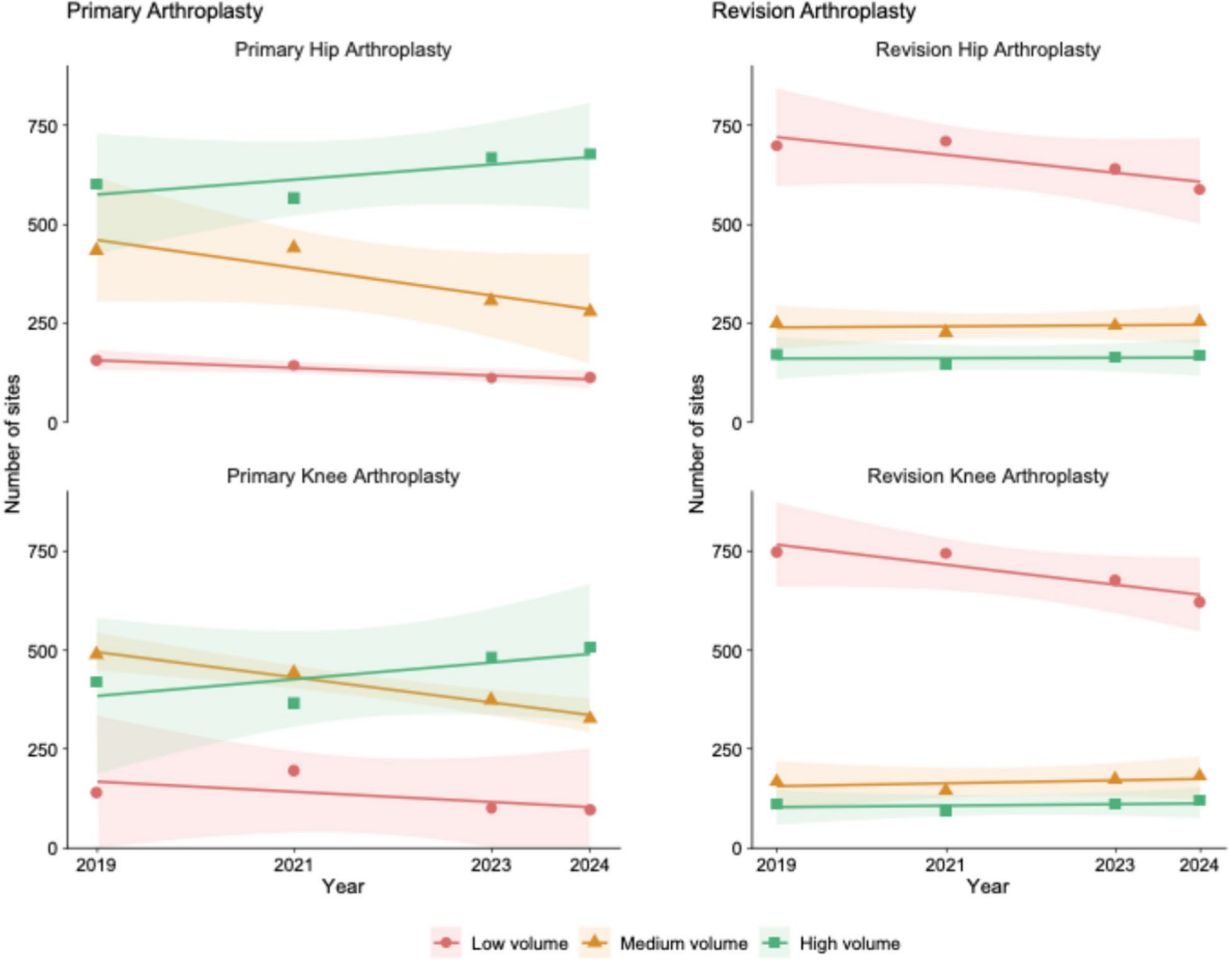
Trends in Hospital Volume Categories Over Time (2019–2024). Observed annual numbers of hospital sites are shown as points. Linear regression lines with 95% confidence intervals (shaded) illustrate trends over time (see also Table 3). Colors indicate site volume categories: red represents low-volume centers, orange represents medium-volume centers, and green represents high-volume centers. For primary arthroplasty, low volume is defined as < 50 procedures per year, medium volume as 50–150, and high volume as > 150. For revision arthroplasty, low volume is defined as < 25 procedures per year, medium volume as 25–50, and high volume as > 50. For further details about the linear model see Table 3

The number of hospitals providing arthroplasties of the hip and knee in Germany differed by volume category and surgery type over time (Fig. 6, Table 3). For revision hip and knee arthroplasty, most hospitals were classified as low-volume centers, indicating that a large proportion of revision procedures were performed in hospitals with relatively small annual case numbers. The number of these low-volume hospitals decreased over time (approximately − 22 and − 25 hospitals per year for revision hip and knee, respectively), although these trends did not reach statistical significance. Medium- and high-volume revision centers remained largely stable, with no meaningful changes over time. In primary arthroplasty, a different pattern was observed. The number of hospitals performing low-volume primary hip arthroplasty decreased significantly by about 10 hospitals per year (p = 0.031), while medium-volume centers also declined numerically. At the same time, high-volume centers for primary hip arthroplasty increased, although this trend was not statistically significant. For primary knee arthroplasty, the number of medium-volume hospitals decreased significantly (approximately − 32 hospitals per year; p = 0.010), whereas low-volume centers remained relatively stable and high-volume centers showed a numerical increase. Overall, these findings suggest a shift of primary arthroplasty procedures toward higher-volume hospitals in Germany, while revision procedures continue to be widely performed in low-volume hospitals despite a gradual reduction in their number.

**Table 3:** Linear regression analysis of hospital site trends

Procedure Type	Volume Group	Intercept	Slope (per year)	95% CI (Slope)	R^2^	p-value
Primary Hip Arthroplasty	Low	20,073	−9.86	[−17.53; −2.19]	0.94	**0.031**
	Medium	70,646	−34.76	[−81.44; 11.92]	0.84	0.085
	High	−37,820	19.02	[−26.39; 64.42]	0.62	0.213
Primary Knee Arthroplasty	Low	25,832	−12.71	[−62.52; 37.10]	0.38	0.387
	Medium	65,274	−32.08	[−46.27; −17.90]	0.98	**0.010**
	High	−43,043	21.51	[−36.99; 80.01]	0.56	0.254
Revision Hip Arthroplasty	Low	45,753	−22.31	[−59.34; 14.73]	0.77	0.122
	Medium	−2,808	1.51	[−14.59; 17.61]	0.08	0.726
	High	−592	0.37	[−15.21; 15.96]	0.01	0.927
Revision Knee Arthroplasty	Low	51,788	−25.27	[−57.03; 6.48]	0.85	0.076
	Medium	−7,545	3.81	[−14.67; 22.29]	0.28	0.468
	High	−4,415	2.24	[−11.02; 15.50]	0.21	0.543

Linear regression analyses were performed to assess temporal trends in the number of hospital sites across procedure types and volume groups (complementary to Fig. 6). The intercept represents the estimated baseline value at year zero, while the slope indicates the annual change in the number of sites. Ninety-five percent confidence intervals (CI) are provided for the slope estimates. Model fit is described by the coefficient of determination (R^2^), and statistical significance is assessed using p-values. Volume groups are defined as low, medium, and high procedural volumes depending on procedure type (primary: < 50 (Low), 50–150 (Medium), > 150 cases (High); revision: < 25 (Low), 25–50 (Medium), > 50 cases (High)

## Discussion

### Summary of key findings

This study demonstrates diverging trends in primary and revision arthroplasty in Germany. While primary hip and knee arthroplasty volumes continue to increase significantly, revision procedures remain stable or show only modest changes, contradicting earlier projections of a parallel rise [2, 3]. At the same time, a clear centralization of primary procedures toward high-volume centres was observed, whereas revision arthroplasty continues to be widely performed in low-volume hospitals. Demographic changes are expected to further shift the burden of arthroplasty toward older patient populations, with a projected increase in procedures among patients over 75 years of age. In parallel, length of hospital stay has decreased substantially across all procedure types, reflecting improved efficiency in perioperative care. Overall, these findings indicate a growing mismatch between increasing demand, evolving patient demographics, and the current structure of care delivery, highlighting the need for targeted adaptations of the healthcare system to ensure adequate and efficient future arthroplasty care.

### Discussion of key findings

The present analysis shows a clear divergence between increasing primary arthroplasty volumes and stable revision rates. Recent large-scale data from the United States confirm this pattern, showing a disproportionate increase in primary procedures compared with revisions, with observed revision volumes substantially lower than expected based on historical failure rates [6, 7]. Similar findings have been reported earlier, with stable or even declining revision burdens despite rising procedure numbers, particularly for hip arthroplasty [8]. Registry-based analyses across multiple countries further support a declining revision burden over time, especially for hip arthroplasty, while knee revision rates remain relatively constant [9, 10]. In contrast, widely cited projections assumed constant revision burdens and therefore substantially overestimated future revision volumes [2, 3, 11]. The most plausible explanation for this decoupling is improved implant survival and a reduction in failure mechanisms such as loosening and wear [6, 12]. In addition, the implementation of national arthroplasty registries has been associated with significant reductions in early revision rates, suggesting a quality improvement effect through feedback and surveillance [13]. Improvements in surgical technique and increasing surgeon experience through rigorous teaching programs may further contribute to declining revision risks [14].

International evidence consistently demonstrates that higher hospital and surgeon volumes are associated with improved outcomes in both primary and revision arthroplasty, including lower complication and revision rates [15]. Specifically for revision procedures, large German database studies show that low-volume hospitals have significantly higher re-revision rates and even increased mortality, strongly supporting centralization of revision care [16, 17]. Despite this, revision arthroplasty remains widely fragmented across low-volume centres, as shown by registry analyses from the UK and US [18, 19]. In contrast, primary arthroplasty has undergone increasing centralization, partly driven by minimum volume regulations and policy initiatives. This mismatch likely reflects missing regulatory frameworks for revision procedures, combined with their lower frequency, higher complexity, insufficient reimbursement and structural barriers to centralization.

The observed continuous reduction in length of hospital stay in Germany is consistent with international trends, although absolute length of hospital stay remains higher compared to countries such as the UK, US, and Scandinavia [20–22]. While length of hospital stay in England and Denmark has already approached one to three days for primary arthroplasty, German data still reflect longer hospitalization but show a clear convergence toward international benchmarks. Importantly, multiple studies demonstrate that these reductions in length of hospital stay are not associated with increased complication or readmission rates, indicating that shorter hospital stays can be achieved safely [20, 21]. The implementation of fast-track or ERAS protocols is a key driver of this development, significantly reducing length of hospital stay without compromising clinical outcomes [5, 23]. However, international comparisons suggest that healthcare system factors play a major role, as Germany’s traditionally longer length of hospital stay is partly explained by structured inpatient rehabilitation pathways and reimbursement models [24, 25]. At the same time, evidence from outpatient arthroplasty indicates that very short stays or same-day discharge can be safe in selected, low-risk patients, but require careful patient selection [26]. Importantly, older patients, revision procedures, and comorbid populations remain associated with longer length of hospital stay and higher complication risks, suggesting a natural limit to further reductions [27]. In this context, the projected continued decrease in length of hospital stay in Germany likely reflects ongoing efficiency gains but must be balanced against patient complexity, quality of care and reimbursement models implemented by current governments.

### Future perspectives

The findings highlight the need for targeted healthcare policy adjustments in Germany. While centralization in primary arthroplasty has progressed, revision care remains fragmented and should be addressed through minimum volume requirements and specialized centre structures. Demographic changes and increasing patient complexity require expansion of high-volume, multidisciplinary centres. In parallel, reimbursement systems must be adapted to support shorter hospital stays and the transition toward outpatient and hybrid care models, that still have to be developed in Germany.

## Data Availability

The data used in this study were obtained from publicly available datasets provided by the German Federal Statistical Office (Destatis) and hospital quality reports of the German Federal Joint Committee (G-BA). Processed data supporting the findings of this study are available from the corresponding author upon reasonable request.
